# Association between air pollution and risk of non-alcoholic fatty liver disease: an updated meta-analysis

**DOI:** 10.3389/fpubh.2025.1606959

**Published:** 2025-08-19

**Authors:** Xu Zhang, Xianzhao Yang, Lanshuo Hu, Lingjie Tan, Xiaoyang Li, Yijie Chai, Shuying Ru

**Affiliations:** ^1^Dongzhimen Hospital, Beijing University of Chinese Medicine, Beijing, China; ^2^Graduate School, Beijing University of Chinese Medicine, Beijing, China; ^3^Tongzhou District of Dongzhimen Hospital, Beijing University of Chinese Medicine, Beijing, China

**Keywords:** air pollution, particulate matter, non-alcoholic fatty liver disease, systematic review, meta-analysis

## Abstract

**Objective:**

Air pollution is a major environmental risk to human health, with increasing evidence linking it to non-alcoholic fatty liver disease (NAFLD). However, findings remain inconsistent. This meta-analysis aimed to assess the relationship between air pollutants and the risk of NAFLD.

**Methods:**

PubMed, Embase, and Web of Science were systematically searched for studies published up to March 20, 2025. A random effects model was used to estimate combined odds ratios (ORs) and 95% confidence intervals (95% CIs). Subgroup analysis, sensitivity analysis, funnel plots, and Egger's test were conducted.

**Results:**

A total of 12 studies, including 49,549,903 participants (published between 2022 and 2024), were analyzed. For each 10 μg/m3 increase in pollutants, the ORs were 1.22 (1.16–1.29) for particulate matter with aerodynamic diameter ≤ 2.5 μm (PM_2.5_), 1.15 (0.95–1.40) for particulate matter between 2.5 and 10 μm in aerodynamic diameter (PM_2.5 − 10_), and 1.07 (1.01–1.13) for particulate matter with aerodynamic diameter ≤ 10 μm (PM_10_). For gaseous pollutants, the ORs were 1.45 (0.92–2.28) for sulfur dioxide (SO_2_) and 1.10 (1.06–1.14) for nitrogen dioxide (NO_2_). No notable connection emerged between ozone (O_3_) or carbon monoxide (CO) and NAFLD. Subgroup analysis revealed stronger associations for PM_2.5_, PM_10_, and NO_2_ with NAFLD in developed countries, Europe, and cohort studies, compared to developing countries, Asia, and cross-sectional studies.

**Conclusion:**

This analysis supports a positive relationship between air pollution and NAFLD risk. Geographic region and economic development appear to moderate this association.

**Systematic review registration:**

https://www.crd.york.ac.uk/PROSPERO/view/CRD42024594146, Identifier: CRD42024594146.

## 1 Introduction

Non-alcoholic fatty liver disease (NAFLD) ranks among the foremost causes of chronic liver conditions globally, encompassing a progression from simple steatosis and steatohepatitis to severe outcomes like fibrosis and cirrhosis (1). Currently affecting 32.4% of people globally, this condition is expected to see a notable surge in prevalence over the next 10 years (2–4). Beyond its toll on liver function and survival, NAFLD is connected to various extrahepatic disorders, such as cardiovascular disease, type 2 diabetes mellitus, chronic kidney disease, and select cancers (5–7), posing a growing public health challenge (4). Understanding the risk factors for NAFLD is crucial for early prevention and management.

Air pollution, a major environmental health threat, contributes to an estimated 7 million premature deaths annually (8) and is linked to chronic diseases including cardiovascular disease, respiratory disorders, and cancer (9–12). Key pollutants include particulate matter (PM), ozone (O_3_), nitrogen dioxide (NO_2_), sulfur dioxide (SO_2_), and carbon monoxide (CO) (13). Recent evidence suggests that air pollutants may also increase NAFLD risk, though findings remain inconsistent (14–16). For instance, some studies have reported a significant association between particulate matter with aerodynamic diameter ≤ 2.5 μm (PM_2.5_) and the risk of NAFLD (17, 18), whereas others have found little to no relationship (19, 20). Experimental studies indicate that the underlying mechanisms may involve oxidative stress, inflammation, and insulin resistance induced by air pollutant exposure (21–23).

A prior meta-analysis (24) focused on NAFLD risk included a limited subset of studies and failed to standardize effect sizes across studies, leading to potential bias. Furthermore, several large, high-quality cohort and cross-sectional studies in recent years have added new insights into the relationship between air pollution and NAFLD risk (17, 20, 25, 26). To address these gaps, we conducted an updated meta-analysis to comprehensively evaluate the relationship between multiple air pollutants (e.g., PM, NO_2_, SO_2_, CO, O_3_) and NAFLD risk, incorporating recent high-quality studies and standardizing effect estimates for improved comparability.

## 2 Methods

This meta-analysis followed the Preferred Reporting Items for Systematic Reviews and Meta-Analyses (PRISMA) guidelines (27) and was registered with PROSPERO (CRD42024594146) before initiation.

### 2.1 Data sources and searches

PubMed, Embase, and Web of Science were explored for pertinent studies released by March 20, 2025. The search was limited to English-language publications and utilized a blend of medical subject headings (MeSH) and terms associated with liver conditions (e.g., “fatty liver,” “steatohepatitis^*^,” “Visceral Steatos^*^,” “liver steatosis^*^”) and atmospheric pollutants (e.g., “air pollution,” “particulate matter,” “nitrogen oxides,” “ozone,” “sulfur dioxide,” “carbon monoxide”). The detailed search strategy is provided in Supplementary Tables S1–S3.

### 2.2 Selection criteria

The meta-analysis included studies that met the following Population, Exposure, Comparison, Outcome, Study Design (PECOS) criteria:

Population: general population, including adults and children.
Exposure: air pollutants, including PM_2.5_, particulate matter between 2.5 and 10 μm in aerodynamic diameter (PM_2.5 − 10_), particulate matter with aerodynamic diameter ≤ 10 μm (PM_10_), NO_2_, SO_2_, CO, and O_3_.
Comparator: non-exposed or less-exposed populations.
Outcome: NAFLD, metabolic-associated fatty liver disease (MAFLD), or metabolic dysfunction-associated steatotic liver disease (MASLD). NAFLD diagnoses were based on ICD-10 codes K75.8 and K76.0, or ultrasonography of hepatic steatosis with complications such as obesity, type 2 diabetes mellitus, or metabolic disorders (MAFLD) (28), or MASLD as defined by hepatic steatosis plus one of five cardiometabolic criteria (29).
Study design: observational studies, including cohort, cross-sectional, and case-control designs.

Studies that reported odds ratios (ORs), risk ratios (RRs), or hazard ratios (HRs) with 95% confidence intervals (CIs), or provided sufficient data for these estimates, were included. Recent or comprehensive studies were prioritized when datasets overlapped (30).

Exclusion criteria included conference abstracts, protocols, reviews, and duplicate publications.

### 2.3 Study selection

Titles and abstracts were independently reviewed by two authors (XZ and LSH) based on pre-set inclusion and exclusion guidelines. Full texts of potentially qualifying studies were then evaluated to confirm their suitability. Any differences of opinion were settled by involving a third author (XZY) as a mediator.

### 2.4 Data extraction

Data were independently gathered by two authors (XZ and LSH), with a third author (XZY) addressing any inconsistencies. Collected details included the first author, year of publication, study design, region, survey period, sample size, mean age, female proportion, air pollutants measured, outcome, statistical model, and effect estimates with corresponding 95% CIs.

### 2.5 Quality assessment

Quality assessments were independently performed by two authors (XZ and LSH). Discrepancies were resolved through discussion. The quality of cohort studies was assessed using the Newcastle-Ottawa Scale (NOS) (31), with scores categorized as: low (0–3), moderate (4–6), or high (7–9) quality. Cross-sectional study quality was assessed using the American Agency for Healthcare Research and Quality criteria (32), with scores classified as: low (0–3), moderate (4–7), or high (8–11).

### 2.6 Statistical analysis

Adjusted ORs and their 95% CIs were relied upon to evaluate the link between air pollutants and NAFLD risk. Studies reporting RRs or HRs were considered equivalent to ORs (33). Pollutant levels reported in parts per billion (ppb) were transformed to μg/m3 using conversion rates: 1.88 for NO_2_, 2.62 for SO_2_, 1.96 for O_3_, and 1.15 for CO. Due to varying increments of air pollutant exposure, ORs and 95% CIs were standardized to a 10 μg/m3 increase using the formula (34):


$$\begin{array}{l} \text{O}{\text{R}}_{\text{(standardized)}}=\text{O}{{\text{R}}_{\text{(original)}}}^{\text{10/Increment(original)}} \end{array}$$

A random-effects model was employed to derive pooled ORs linking NAFLD with pollutant exposure. Heterogeneity was examined using Cochran's *Q* test and the *I*^2^ metric, where *I*^2^ exceeding 50% denoted substantial variability. Subgroup analyses explored heterogeneity sources, considering factors like study type, sample size, female ratio, economic context, smoking prevalence, region, education, and outcome classification. Sensitivity tests verified the stability of results. Funnel plots and Egger's regression test were used to detect publication bias (35), with a minimum of 10 studies required for this analysis (36). Statistical analyses were performed using Stata version 14.0.

## 3 Results

### 3.1 Literature selection

**Figure 1:**
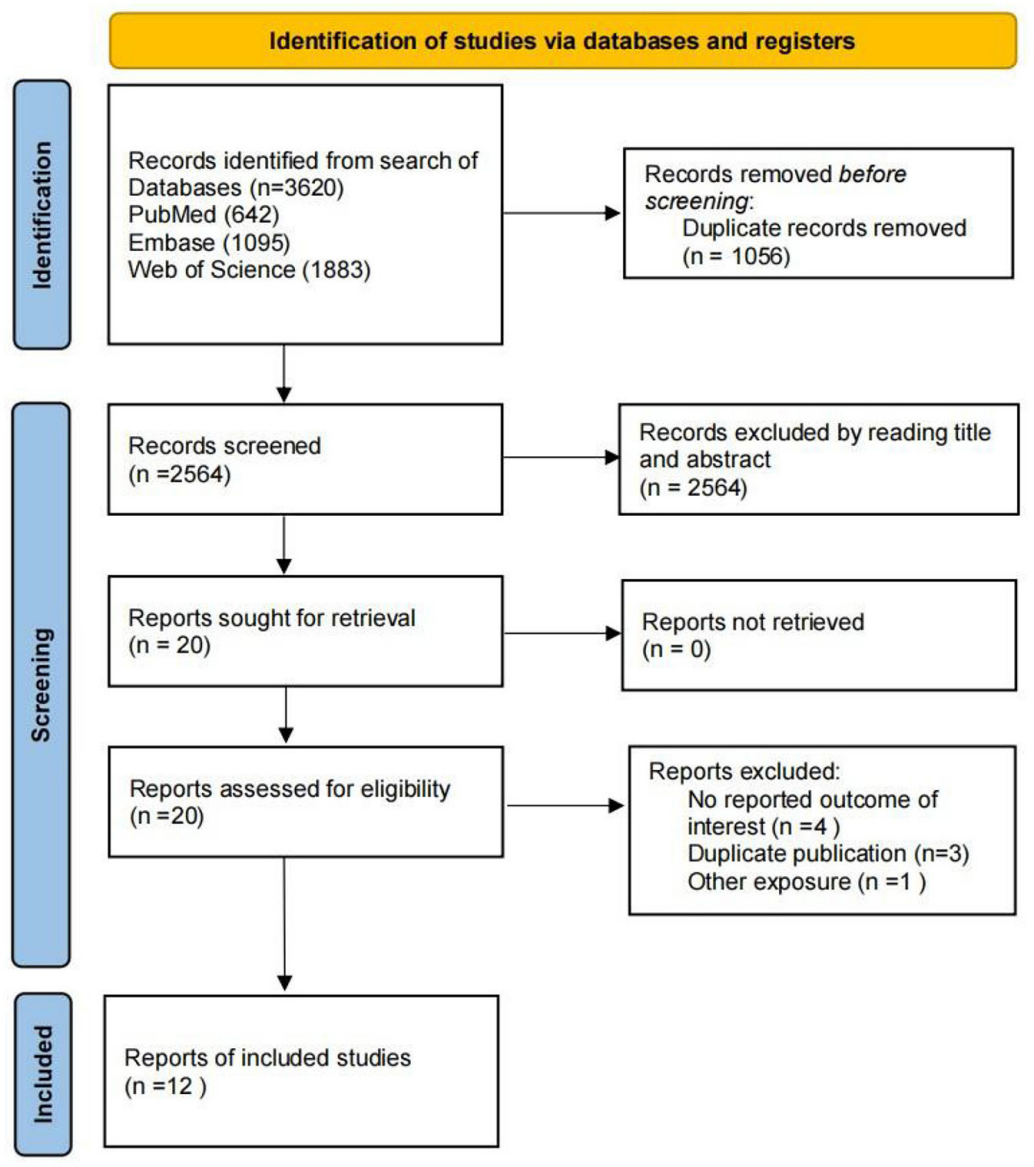
Literature screening flowchart.

The search strategy identified 3,620 relevant articles. After removing duplicates, 2,564 records underwent title and abstract evaluation. Full-text assessment was performed on 20 articles, of which eight were excluded: four for lack of relevant results, three for duplication, and one for addressing a different exposure. Ultimately, 12 studies were retained for the meta-analysis (Figure 1).

### 3.2 Study characteristics

The 12 included studies (14–18, 20, 25, 26, 37–40), published between 2022 and 2024, involved a total of 49,549,903 participants. Of these, seven were cohort studies (17, 18, 20, 25, 37, 39, 40) and five were cross-sectional (14–16, 26, 38). Sample sizes ranged from 170 to 45,433,392. Studies focused on various air pollutants: 12 on PM_2.5_, three on PM_2.5 − 10_, seven on PM_10_, three on SO_2_, eight on NO_2_, five on O_3_, and three on CO. Geographically, seven studies were conducted in Asia, three in Europe, and two in North America. Confounders adjusted for in the studies varied slightly. Quality assessment revealed eight studies rated as high quality and four as medium quality (Supplementary Tables S4, S5). Detailed study characteristics are provided in Table 1.

**Table 1:** Characteristics of studies included in the meta-analysis.

**Study ID**	**Study type**	**Region**	**Survey time**	**Sample size (Case/Control)**	**Age (Mean ±SD, years)**	**Female ratio**	**Pollutant**	**Outcome**	**Statistical model**	**Quality score**
Kong 2024	Prospective cohort	UK	2006–2010	417,250 (4,752/412,273)	56.3 ± 8.1	223,892 (53.7)	PM_2.5_, PM_2.5 − 10_, PM_10_, NO_2_	NAFLD	Cox proportional hazard model	7
Feng 2024	Retrospective cohort	China	3 years preceding outcome assessment	27,699 (7,374/20,325)	NA	4,052 (14.6)	PM_2.5_, SO_2_, NO_2_, O_3_, CO	MAFLD	Logistic regression model	6
Bo 2024	Cross-sectional	China	2001–2018	329,048 (96,852/232,196)	41.0 ± 13.0	185,164 (56.3)	PM_2.5_, NO_2_, O_3_	NAFLD	Logistic regression model	8
Ji 2024	Cross-sectional	China	2017–2020	2,711,207 (191,592/2,519,615)	49.7 ± 15.4	1,432,694 (52.8)	PM_2.5_, PM_10_, SO_2_, O_3_, CO	MAFLD	Spatial generalized linear mixed models	7
Cheng 2024	Cross-sectional	China	2010–2017	131,592 (53,431/78,161)	NA	68,396 (52.0)	PM_2.5_, PM_10_, SO_2_, NO_2_, O_3_, CO	MASLD	Logistic regression model	7
Patterson 2023	Prospective cohort	US	2014–2018	170 (30/110)	19.7 ± 1.2	56 (45.2)	PM_2.5_, PM_10_, NO_2_, O_3_	NAFLD	Logistic regression model	7
Han 2023	Longitudinal cohort	China	2018–2019	6,350 (744/5,786)	NA	4,145 (63.5)	PM_2.5_, PM_10_, NO_2_	MAFLD	Multiple logistic regression models	7
Matthiessen 2023	Cross-sectional	Germany	2000–2003	4,065 (1,288/2,777)	59.6 ± 7.8	2,157 (53.1)	PM_2.5_, PM_2.5 − 10_, PM_10_, NO_2_	NAFLD	Logistic regression model	8
Zhao 2023	Prospective cohort	China	June 2011–December 2013	15,337 (1,516/13,821)	47.6 ± 7.9	6,914 (45.1)	PM_2.5_	MAFLD	Cox proportional hazard model	7
Deng 2023	Population-based dynamic cohort	China	2005–2013	17,106 (4,640/12,466)	NA	12,231 (71.5)	PM_2.5_	NAFLD	Cox proportional hazard model	8
Li 2022	Prospective cohort	UK	2006–2010	456,687 (4,978/451,709)	NA	NA	PM_2.5_, PM_2.5 − 10_, PM_10_, NO_2_	NAFLD	Cox proportional hazard model	8
VoPham 2022	Cross-sectional	US	2001–2011	45,433,392 (269,705/45,163,687)	48.6 ± 28.0	26,550,788 (58.4)	PM_2.5_	NAFLD	Multivariable logistic regression model	6

PM_2.5_, particulate matter with aerodynamic diameter ≤ 2.5 μm; PM_2.5 − 10_, particulate matter between 2.5 and 10 μm in aerodynamic diameter; PM_10_, particulate matter with aerodynamic diameter ≤ 10 μm; NO_2_, nitrogen dioxide; O_3_, ozone; SO_2_, sulfur dioxide; UK, United Kingdom; US, United States; SD: standard deviation; NA, not available.

### 3.3 Exposure of air pollution and NAFLD

**Figure 2:**
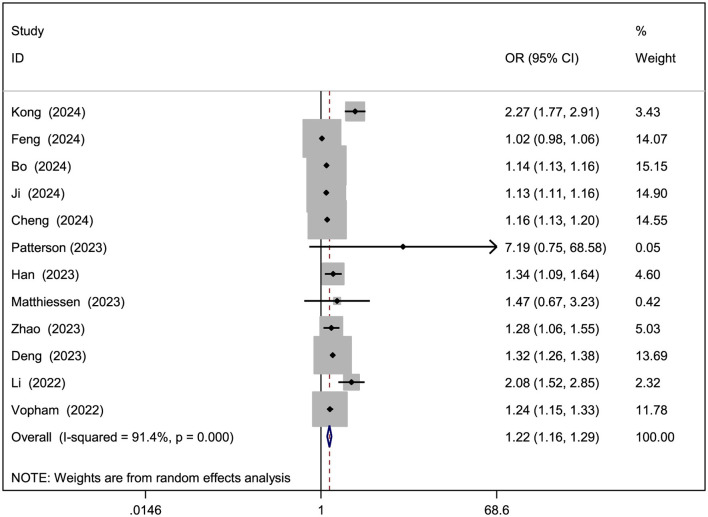
Forest plot of the association between PM_2.5_ exposure (per 10 μg/m^3^ increment) and risk of NAFLD.

**Figure 3:**
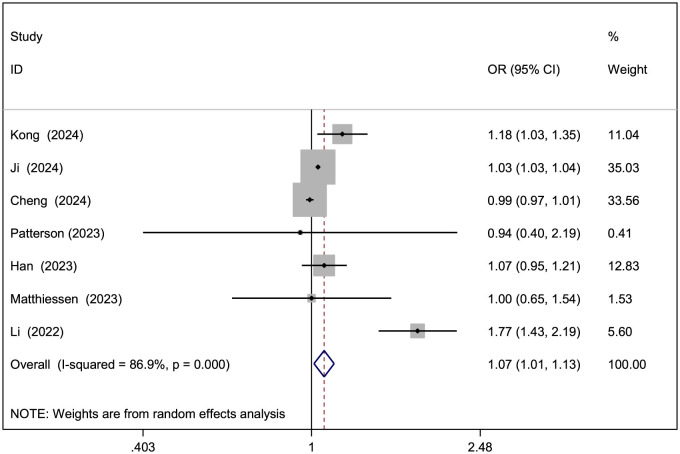
Forest plot of the association between PM_10_ exposure (per 10 μg/m^3^ increment) and risk of NAFLD.

**Figure 4:**
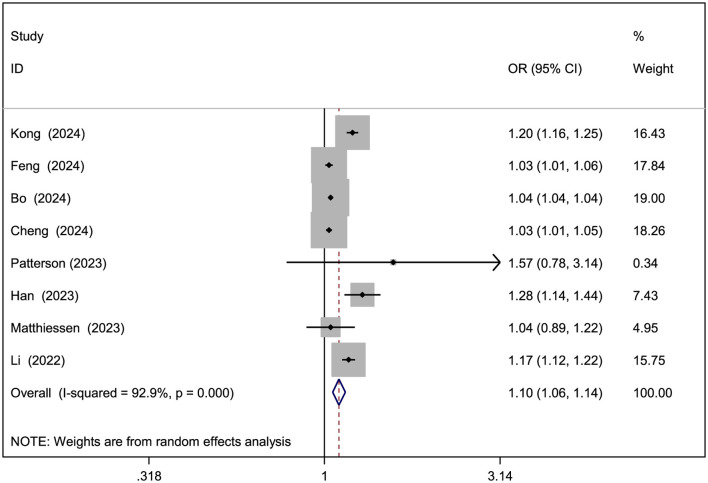
Forest plot of the association between NO_2_ exposure (per 10 μg/m^3^ increment) and risk of NAFLD.

Table 2 presents the ORs linking air pollutants to NAFLD, with forest plots for analyses involving over five studies shown in Figures 2–4. A 10 μg/m3 rise in PM_2.5_, PM_10_, and NO_2_ levels was notably tied to elevated NAFLD risk (PM_2.5_ OR = 1.22, 95% CI: 1.16–1.29; PM_10_ OR = 1.07, 95% CI: 1.01–1.13; NO_2_ OR = 1.10, 95% CI: 1.06–1.14; Figures 2–4). However, significant heterogeneity was observed across studies (PM_2.5_ *I*^2^ = 91.4%, *P* < 0.001; PM_10_ *I*^2^ = 86.9%, *P* < 0.001; NO_2_ *I*^2^ = 92.9%, *P* < 0.001). For PM_2.5 − 10_ and SO_2_, a 10 μg/m3 increase suggested increased NAFLD likelihood, though these findings did not achieve statistical significance (PM_2.5 − 10_ OR = 1.15, 95% CI: 0.95–1.40, *P* = 0.15; SO_2_ OR = 1.45, 95% CI: 0.92–2.28, *P* = 0.113), with SO_2_ showing marked heterogeneity (*I*^2^ = 98.4%, *P* < 0.001). No notable connection emerged between O_3_ or CO and NAFLD.

**Table 2:** Meta-analysis of NAFLD in association with a 10 μg/m^3^ increase in PM_2.5_, PM_2.5 − 10_, PM_10_, SO_2_, NO_2_, O_3_, and CO.

**Overall analysis**	**Pooled OR (95% CIs)**	***P* value**	**No. of studies**	**Heterogeneity**	
				***P* value**	***I* ^2^**
PM_2.5_	1.22 (1.16, 1.29)	<0.001	12	<0.001	91.4%
PM_2.5 − 10_	1.15 (0.95, 1.40)	0.150	3	0.937	0.0%
PM_10_	1.07 (1.01, 1.13)	0.016	7	<0.001	86.9%
SO_2_	1.45 (0.92, 2.28)	0.113	3	<0.001	98.4%
NO_2_	1.10 (1.06, 1.14)	<0.001	8	<0.001	92.9%
O_3_	1.01 (0.90, 1.13)	0.837	5	<0.001	98.7%
CO	1.02 (0.99, 1.05)	0.281	3	<0.001	99.4%

### 3.4 Subgroup analysis

Given the scarcity of studies, subgroup analyses were not feasible for PM_2.5 − 10_, SO_2_, O_3_, or CO. Subgroup analyses for PM_2.5_, PM_10_, and NO_2_ are presented in Tables 3–5. In most subgroups, exposure to these pollutants remained positively associated with NAFLD, although heterogeneity remained at moderate to high levels. Stronger associations were observed in studies from developed countries, Europe, and cohort studies compared to those from developing countries, Asia, and cross-sectional studies. Specifically, a significant and robust association was found for PM_2.5_ in Europe (OR = 2.15, 95% CI: 1.78–2.59, *P* < 0.001, *I*^2^ = 0%). Stratified analysis of PM_10_ in developed countries, with sample sizes < 100,000, showed *I*^2^ = 0%, as did stratified analysis of NO_2_ in cross-sectional studies.

**Table 3:** Association between exposure to PM_2.5_ and risk of NAFLD by subgroup analysis.

**Subgroups**	**Categories**	**No. of studies**	**OR, 95%CI**	***I*^2^ (%)**	***P* values within subgroups**
Study design	Cross-sectional	5	1.15 (1.13, 1.17)	48.5	<0.001
	Cohort	7	1.45 (1.21, 1.74)	95.0	<0.001
Sample size	< 100,000	6	1.25 (1.04, 1.49)	93.5	0.015
	>100,000	6	1.20 (1.15, 1.27)	90.0	<0.001
Female, %	< 50%	3	1.15 (0.89, 1.49)	75.3	0.275
	>50%	9	1.25 (1.18, 1.31)	90.9	<0.001
Economic level	Developed countries	4	1.72 (1.05, 2.82)	87.2	0.031
	Developing countries	8	1.18 (1.12, 1.24)	92.2	<0.001
Current smoker, %	< 25%	7	1.22 (1.15, 1.29)	86.8	<0.001
	>25%	2	1.12 (0.90, 1.39)	81.0	0.310
Geographic area	Asia	7	1.16 (1.11, 1.22)	92.1	<0.001
	Europe	3	2.15 (1.78, 2.59)	0.0	<0.001
College or above	< 50%	3	1.20 (1.08, 1.34)	51.4	0.001
	>50%	5	1.16 (1.08, 1.24)	94.6	<0.001
Outcome	NAFLD	7	1.42 (1.25, 1.61)	93.0	<0.001
	MAFLD	4	1.14 (1.04, 1.24)	88.8	0.006

**Table 4:** Association between exposure to PM_10_ and risk of NAFLD by subgroup analysis.

**Subgroups**	**Categories**	**No. of studies**	**OR, 95%CI**	***I*^2^ (%)**	***P* values within subgroups**
Study design	Cross-sectional	3	1.01 (0.97, 1.06)	87.7	0.542
	Cohort	4	1.26 (1.00, 1.60)	82.2	0.053
Sample size	< 100,000	3	1.06 (0.95, 1.19)	0.0	0.305
	>100,000	4	1.07 (1.01, 1.14)	93.4	0.021
Economic level	Developed countries	3	1.16 (1.02, 1.31)	0.0	0.025
	Developing countries	4	1.06 (1.00, 1.12)	92.8	0.058
Geographic area	Asia	3	1.02 (0.98, 1.06)	88.0	0.380
	Europe	3	1.31 (0.95, 1.81)	82.8	0.097
Outcome	NAFLD	4	1.27 (0.95, 1.71)	75.4	0.107
	MAFLD	2	1.03 (1.03, 1.04)	0.0	<0.001

**Table 5:** Association between exposure to NO_2_ and risk of NAFLD by subgroup analysis.

**Subgroups**	**Categories**	**No. of studies**	**OR,95%CI**	**I^2^ (%)**	***P* values within subgroups**
Study design	Cross-sectional	3	1.04 (1.03, 1.04)	0.0	<0.001
	Cohort	5	1.16 (1.06, 1.28)	93.8	0.002
Sample size	< 100,000	4	1.12 (0.97, 1.29)	78.7	0.110
	>100,000	4	1.01 (1.04, 1.17)	96.5	<0.001
Economic level	Developed countries	3	1.16 (1.03, 1.30)	44.3	0.016
	Developing countries	5	1.07 (1.04, 1.11)	90.6	<0.001
Geographic area	Asia	4	1.04 (1.02, 1.07)	78.0	<0.001
	Europe	3	1.18 (1.13, 1.23)	40.8	<0.001
Outcome	NAFLD	5	1.12 (1.02, 1.24)	95.3	0.015
	MAFLD	2	1.14 (0.92, 1.41)	92.2	0.229

### 3.5 Sensitivity analysis and publication bias

**Figure 5:**
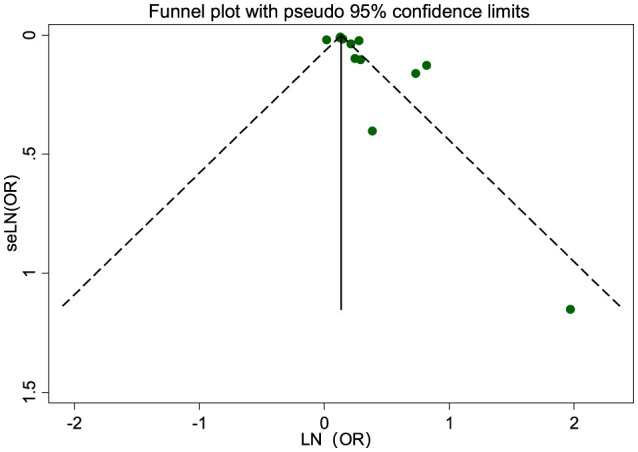
Funnel plot of the association between PM_2.5_ exposure (per 10 μg/m^3^ increment) and risk of NAFLD.

Sensitivity analysis, conducted by excluding one study at a time, indicated that the results for most air pollutants were stable (Supplementary Figure S1). Publication bias tests were not conducted for exposures with fewer than 10 studies. Visual inspection of the funnel plot for PM_2.5_ indicated no significant publication bias (Figure 5). Additionally, Egger's regression test (*P* = 0.069) supported the absence of publication bias.

## 4 Discussion

Based on 12 studies involving 49,549,903 participants, the results indicate that exposure to air pollutants, including PM_2.5_, PM_2.5 − 10_, PM_10_, SO_2_, NO_2_, O_3_, and CO, correlates with an increased risk of NAFLD. However, considerable heterogeneity was observed across studies, with the associations for PM_2.5 − 10_ and SO_2_ not reaching statistical significance. Subgroup and sensitivity analyses supported these overall findings, with consistent associations observed across most subgroups.

A prior meta-analysis by He et al. reported that PM_2.5_, NO_x_, PM_10_, and PM_2.5 − 10_ elevate NAFLD risk, while suggesting a protective role for O_3_ (24). That review, however, was limited to a subset of earlier studies on air pollution and MAFLD risk, introducing potential selection bias. In addition, the meta-analysis did not normalize effect sizes to the same increments across studies. In contrast, this work involved an exhaustive review of research linking air pollution to NAFLD, MAFLD, or MASLD, included several recent, large-sample, high-quality studies, and standardized effect sizes to 10 μg/m3 increments. Therefore, this research has more advantages in timeliness and comparability. In addition, instead of including the cross-sectional study by Guo et al. (41), the same sample, the latest cohort study by Han et al. (25), was chosen, which contributes to the reliability of causal inference. Notably, unlike the results from He et al., no significant association was found between O_3_ and NAFLD. Stronger associations were observed between PM_2.5_, PM_10_, NO_2_, and NAFLD in developed countries, particularly in Europe, and in cohort studies.

Subgroup analysis indicated a stronger association between PM_2.5_, PM_10_, and NO_2_ with NAFLD in cohort studies, which offer greater reliability for inferring correlation compared to cross-sectional studies. Additionally, a more pronounced and statistically significant association was found for PM_2.5_ in women over 50%. This may be linked to sex-specific differences in susceptibility to air pollution, as previous studies have suggested women may have a higher susceptibility to respiratory and cardiovascular issues related to PM_2.5_ exposure (42, 43). Furthermore, estrogen has been shown to regulate liver lipid metabolism, potentially offering protective effects in women (44–47). The decline in estrogen levels post-menopause may increase susceptibility to NAFLD in women exposed to air pollution, contributing to the observed gender differences. Gender differences in NAFLD risk may, therefore contribute to heterogeneity in the results. Regional variations were also evident, with stronger associations found in studies from developed countries, particularly Europe, for PM_2.5_ and NO_2_, possibly reflecting economic and environmental influences—though limited study numbers constrain broader conclusions.

In stratified analysis comparing NAFLD and MAFLD outcomes, no significant differences were observed regarding their associations with PM_2.5_, PM_10_, and NO_2_. Notably, a study by the European LITMUS “Liver Investigation: Testing Marker Utility in Steatohepatitis” Consortium found that 98% of NAFLD cases met the MASLD criteria (29), and data from Song et al. indicated that most NAFLD patients met the metabolic criteria for MAFLD and MASLD (48). This suggests that findings for NAFLD are applicable under both the MAFLD and MASLD definitions.

The mechanisms by which air pollution contributes to NAFLD remain incompletely understood, though animal studies offer valuable insights. Chronic exposure to PM_2.5_ has been demonstrated to cause liver inflammation, oxidative stress, and insulin resistance, which are pivotal factors in the pathogenesis of NAFLD (49–52). Experimental studies also suggest that PM_2.5_ disrupts liver glycogen storage, causes glucose intolerance, and contributes to non-alcoholic steatohepatitis (53, 54). Furthermore, PM_2.5_ exposure activates hepatic stellate cells, promoting liver fibrosis (55, 56). NO_2_ and O_3_ exposure may also impair lipid metabolism and trigger insulin resistance, potentially exacerbating NAFLD risk (57–59). However, this analysis did not show a significant correlation between O_3_ and NAFLD, possibly due to differences in species, exposure levels, or study design. The effects of SO_2_ and CO on NAFLD remain unclear and warrant further research.

Given the global health burden of air pollution and rising NAFLD prevalence, our findings highlight the urgent need for improved air quality, which may help prevent NAFLD and other related non-communicable diseases, like cardiovascular and respiratory conditions (60, 61).

This study has several limitations. First, methodological and clinical heterogeneity was observed due to variations in confounder adjustment and study designs. Although subgroup analyses were conducted to explore potential sources of heterogeneity, the limited number of studies restricted a comprehensive identification of contributing factors. Second, the analysis focused on individual pollutants, despite the potential for combined effects of air pollutants on health outcomes. The complexity of joint effects and the scarcity of relevant studies precluded their inclusion in this meta-analysis. Third, the included studies did not consistently stratify NAFLD by severity (e.g., simple steatosis vs. non-alcoholic steatohepatitis or fibrosis), limiting the ability to assess the impact of air pollution on non-reversible forms of the disease. Future studies stratifying outcomes by NAFLD severity could reduce heterogeneity and provide clearer insights into the role of air pollution. Finally, given the heterogeneity and limited number of studies for certain pollutants, caution is warranted when interpreting the findings. Larger-scale cohort studies are needed to validate these results and further elucidate the relationship between air pollution and NAFLD risk.

## 5 Conclusion

This meta-analysis provides evidence that exposure to air pollutants, particularly PM_2.5_, PM_10_, and NO_2_, is associated with an increased risk of NAFLD. These findings underscore the importance of improving air quality to mitigate the burden of NAFLD and related diseases. However, it remains to be determined whether air pollutants directly target the liver or contribute to NAFLD by aggravating obesity and insulin resistance in air-polluted environments. Future research should focus on large-scale, longitudinal cohort studies that stratify NAFLD by severity, evaluate the combined effects of multiple air pollutants, and explore underlying biological mechanisms.

## Data Availability

The original contributions presented in the study are included in the article/Supplementary material, further inquiries can be directed to the corresponding authors.
