# Supplementary material for: Association between air pollution and risk of non-alcoholic fatty liver disease: an updated meta-analysis
Source: Front Public Health. 2025 Aug 19;13:1606959. doi: 10.3389/fpubh.2025.1606959 (PMC12401902; doi:10.3389/fpubh.2025.1606959)
Supplement: Supplementary file 1 [file Table_1.docx]

**Supplementary**

**The retrieval strategies and retrieval results of each database are shown in Table S1-3**.

**Table S1.** PubMed (2025.3.20)

| No. | Content | Result |
| --- | --- | --- |
| #1 | Search: ((((("Air Pollution"[Mesh]) OR "Air Pollutants"[Mesh]) OR "Particulate Matter"[Mesh]) OR "Nitrogen Oxides"[Mesh]) OR "Ozone"[Mesh]) OR "Sulfur Dioxide"[Mesh] Sort by: Most Recent | 304,506 |
| #2 | Search: ((((((((((((((Air Pollutant*[Title/Abstract]) OR (Air Pollution*[Title/Abstract])) OR (Particulate Matter*[Title/Abstract])) OR (Nitrogen Oxide*[Title/Abstract])) OR (Ozone*[Title/Abstract])) OR (fine partic*[Title/Abstract])) OR (Coarse particl*[Title/Abstract])) OR (ultrafine particl*[Title/Abstract])) OR (particle number concentration[Title/Abstract])) OR (carbon monoxide[Title/Abstract])) OR (Black carbon[Title/Abstract])) OR (PM2.5[Title/Abstract])) OR (PM10[Title/Abstract])) OR (nitrogen dioxide[Title/Abstract])) OR (nitric oxide[Title/Abstract]) Sort by: Most Recent | 312,328 |
| #3 | Search: (((((("Air Pollution"[Mesh]) OR "Air Pollutants"[Mesh]) OR "Particulate Matter"[Mesh]) OR "Nitrogen Oxides"[Mesh]) OR "Ozone"[Mesh]) OR "Sulfur Dioxide"[Mesh]) OR (((((((((((((((Air Pollutant*[Title/Abstract]) OR (Air Pollution*[Title/Abstract])) OR (Particulate Matter*[Title/Abstract])) OR (Nitrogen Oxide*[Title/Abstract])) OR (Ozone*[Title/Abstract])) OR (fine partic*[Title/Abstract])) OR (Coarse particl*[Title/Abstract])) OR (ultrafine particl*[Title/Abstract])) OR (particle number concentration[Title/Abstract])) OR (carbon monoxide[Title/Abstract])) OR (Black carbon[Title/Abstract])) OR (PM2.5[Title/Abstract])) OR (PM10[Title/Abstract])) OR (nitrogen dioxide[Title/Abstract])) OR (nitric oxide[Title/Abstract])) Sort by: Most Recent | 462,648 |
| #4 | Search: "Fatty Liver"[Mesh] Sort by: Most Recent | 53,373 |
| #5 | Search: ((((Fatty liver*[Title/Abstract]) OR (Steatohepatiti*[Title/Abstract])) OR (Visceral Steatos*[Title/Abstract])) OR (steatohepatitis*[Title/Abstract])) OR (liver steatosis*[Title/Abstract]) Sort by: Most Recent | 64,475 |
| #6 | Search: ("Fatty Liver"[Mesh]) OR (((((Fatty liver*[Title/Abstract]) OR (Steatohepatiti*[Title/Abstract])) OR (Visceral Steatos*[Title/Abstract])) OR (steatohepatitis*[Title/Abstract])) OR (liver steatosis*[Title/Abstract])) Sort by: Most Recent | 78,116 |
| #7 | Search: ((((((("Air Pollution"[Mesh]) OR "Air Pollutants"[Mesh]) OR "Particulate Matter"[Mesh]) OR "Nitrogen Oxides"[Mesh]) OR "Ozone"[Mesh]) OR "Sulfur Dioxide"[Mesh]) OR (((((((((((((((Air Pollutant*[Title/Abstract]) OR (Air Pollution*[Title/Abstract])) OR (Particulate Matter*[Title/Abstract])) OR (Nitrogen Oxide*[Title/Abstract])) OR (Ozone*[Title/Abstract])) OR (fine partic*[Title/Abstract])) OR (Coarse particl*[Title/Abstract])) OR (ultrafine particl*[Title/Abstract])) OR (particle number concentration[Title/Abstract])) OR (carbon monoxide[Title/Abstract])) OR (Black carbon[Title/Abstract])) OR (PM2.5[Title/Abstract])) OR (PM10[Title/Abstract])) OR (nitrogen dioxide[Title/Abstract])) OR (nitric oxide[Title/Abstract]))) AND (("Fatty Liver"[Mesh]) OR (((((Fatty liver*[Title/Abstract]) OR (Steatohepatiti*[Title/Abstract])) OR (Visceral Steatos*[Title/Abstract])) OR (steatohepatitis*[Title/Abstract])) OR (liver steatosis*[Title/Abstract]))) Sort by: Most Recent | 642 |

**Table S2.** Embase

| No. | Content | Result |
| --- | --- | --- |
| #1 | 'air pollution'/exp OR 'air pollutants'/exp OR 'particulate matter'/exp OR 'nitrogen oxides'/exp OR 'ozone'/exp OR 'sulfur dioxide'/exp | 293,010 |
| #2 | 'air pollutant*':ab,ti OR 'air pollution*':ab,ti OR 'particulate matter*':ab,ti OR 'nitrogen oxide*':ab,ti OR ozone*:ab,ti OR 'fine partic*':ab,ti OR 'ultrafine particl*':ab,ti OR 'particle number concentration':ab,ti OR 'carbon monoxide':ab,ti OR 'black carbon':ab,ti OR pm2.5:ab,ti OR pm10:ab,ti OR 'nitrogen dioxide':ab,ti OR 'nitric oxide':ab,ti | 381,955 |
| #3 | #1 OR #2 | 561,200 |
| #4 | 'fatty liver'/exp | 137,203 |
| #5 | 'fatty liver*':ab,ti OR steatohepatiti*:ab,ti OR 'visceral steatos*':ab,ti OR steatohepatitis*:ab,ti OR 'liver steatosis*':ab,ti | 97,442 |
| #6 | #4 OR #5 | 147,708 |
| #7 | #3 AND #6 | 1,095 |

**Table S3.** Web of science

| No. | Content | Result |
| --- | --- | --- |
| #1 | ((((((((((((((((((((TS=(Air Pollution)) OR TS=(Air Pollutants)) OR TS=(Particulate Matter)) OR TS=(Nitrogen Oxides)) OR TS=(Ozone)) OR TS=(Sulfur Dioxide)) OR TS=(Sulfur Dioxide)) OR TS=(Air Pollution*)) OR TS=(Particulate Matter*)) OR TS=(Nitrogen Oxide*)) OR TS=(Ozone*)) OR TS=(fine partic*)) OR TS=(Coarse particl*)) OR TS=(ultrafine particl*)) OR TS=(particle number concentration)) OR TS=(carbon monoxide)) OR TS=(Black carbon )) OR TS=(PM2.5)) OR TS=(PM10)) OR TS=(nitrogen dioxide )) OR TS=(nitric oxide ) | 809,478 |
| #2 | (((((TS=(Fatty Liver)) OR TS=(Fatty liver*)) OR TS=(Steatohepatiti*)) OR TS=(Visceral Steatos*)) OR TS=(steatohepatitis*)) OR TS=(liver steatosis*) | 115,374 |
| #3 | #1 AND #2 | 1,883 |

**Table S4.** The quality assessment of cohort studies.

| Study ID | Selection | Comparability | Outcome | Total |
| --- | --- | --- | --- | --- |
| Cohort studies (n=7) | | | | |
| Kong 2024 | *** | ** | ** | 7 |
| Feng 2024 | *** | ** | * | 6 |
| Patterson 2023 | **** | ** | * | 7 |
| Han 2023 | **** | ** | * | 7 |
| Zhao 2023 | *** | ** | ** | 7 |
| Deng 2023 | **** | ** | ** | 8 |
| Li 2022 | **** | ** | ** | 8 |

The NOS scale was used to evaluate the quality of the cohort studies.

**Table S5.** Quality assessment of cross-sectional studies included.

| **Item Yes/No/Unclear** | **Author, year** | | | | |
| --- | --- | --- | --- | --- | --- |
|  | Bo 2024 | Ji 2024 | Cheng 2024 | Matthiesse 2023 | VoPham 2022 |
| Define the source of information (survey, record review) | Yes | Yes | Yes | Yes | Yes |
| List inclusion and exclusion criteria for exposed and unexposed subjects (cases and controls) or refer to previous publications | Yes | Yes | Yes | Yes | No |
| Indicate time period used for identifying patients | Yes | Yes | Yes | Yes | Yes |
| Indicate whether or not subjects were consecutive if not population-based | Yes | Yes | Yes | Yes | Yes |
| Indicate if evaluators of subjective components of study were masked to other aspects of the status of the participants | No | No | No | No | No |
| Describe any assessments undertaken for quality assurance purposes (e.g., test/retest of primary outcome measurements) | Yes | Yes | Yes | Yes | Yes |
| Explain any patient exclusions from analysis | Yes | No | No | Yes | No |
| Describe how confounding was assessed and/or controlled | Yes | Yes | Yes | Yes | Yes |
| If applicable, explain how missing data were handled in the analysis | No | No | No | No | No |
| Summarize patient response rates and completeness of data collection | No | No | No | No | No |
| Clarify what follow-up, if any, was expected and the percentage of patients for which incomplete data or follow-up was obtained | No | No | No | No | No |
| **Score** | 8 | 7 | 7 | 8 | 6 |

The cross-sectional studies were assessed by the Agency for Healthcare Research and Quality (AHRQ) checklist.


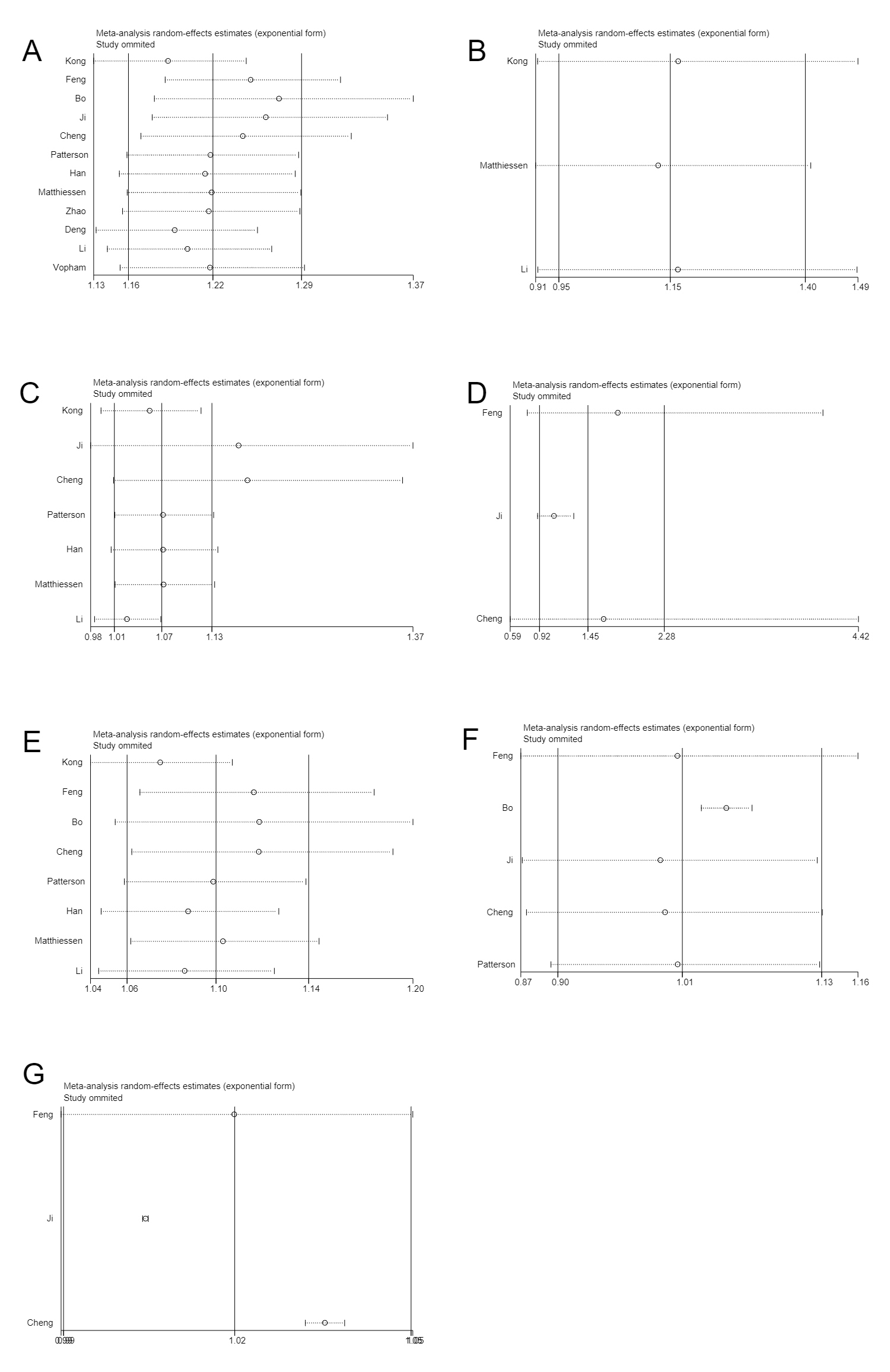


**Figure S1.** Sensitivity analysis results of (A) PM_2.5_, (B) PM_2.5-10_, (C) PM_10_, (D) SO_2_, (E) NO_2_, (F) O_3_, and (G) CO.
